# The Oxford Face Matching Test: A non-biased test of the full range of individual differences in face perception

**DOI:** 10.3758/s13428-021-01609-2

**Published:** 2021-06-15

**Authors:** Mirta Stantic, Rebecca Brewer, Bradley Duchaine, Michael J. Banissy, Sarah Bate, Tirta Susilo, Caroline Catmur, Geoffrey Bird

**Affiliations:** 1 Department of Experimental Psychology, University of Oxford, New Radcliffe House, Walton St, Oxford, OX2 6BW UK; 2 Department of Psychology, Royal Holloway University of London, Egham, TW20 0EY UK; 3 Department for Psychological and Brain Sciences, Dartmouth College, 6207 Moore Hall, Hanover, NH USA; 4 Department of Psychology, Goldsmiths University of London, New Cross, London, SE14 6NW UK; 5 School of Psychological Science, University of Bristol, Bristol, BS8 1TU UK; 6 Department of Psychology, Bournemouth University, Fern Barrow, Wallisdown, Poole, BH12 5BB UK; 7 School of Psychology, Victoria University of Wellington, Kelburn Parade, Wellington, New Zealand; 8 Department of Psychology, King’s College London, London, SE1 9RT UK; 9 Social, Genetic and Developmental Psychiatry Centre, Institute of Psychiatry, Psychology and Neuroscience, King’s College London, SE5 8AF, London, UK

**Keywords:** Oxford Face Matching Test, Cambridge Face Memory Test, Prosopagnosia, Super recogniser, Face perception, Face memory

## Abstract

Tests of face processing are typically designed to identify individuals performing outside of the typical range; either prosopagnosic individuals who exhibit poor face processing ability, or super recognisers, who have superior face processing abilities. Here we describe the development of the Oxford Face Matching Test (OFMT), designed to identify individual differences in face processing across the full range of performance, from prosopagnosia, through the range of typical performance, to super recognisers. Such a test requires items of varying difficulty, but establishing difficulty is problematic when particular populations (e.g., prosopagnosics, individuals with autism spectrum disorder) may use atypical strategies to process faces. If item difficulty is calibrated on neurotypical individuals, then the test may be poorly calibrated for atypical groups, and vice versa. To obtain items of varying difficulty, we used facial recognition algorithms to obtain face pair similarity ratings that are not biased towards specific populations. These face pairs were used as stimuli in the OFMT, and participants were required to judge whether the face images depicted the same individual or different individuals. Across five studies the OFMT was shown to be sensitive to individual differences in the typical population, and in groups of both prosopagnosic individuals and super recognisers. The test-retest reliability of the task was at least equivalent to the Cambridge Face Memory Test and the Glasgow Face Matching Test. Furthermore, results reveal, at least at the group level, that both face perception and face memory are poor in those with prosopagnosia, and are good in super recognisers.

**Supplementary Information:**

The online version contains supplementary material available at 10.3758/s13428-021-01609-2.

The ability to recognise our conspecifics accurately and rapidly is critical in supporting our social interactions. Impaired recognition of others is thought to contribute to social anxiety (Yardley et al., 2008; Dalrymple et al., 2014a), and to trigger a cascade of impairments including in the inference of mental states (Conway et al., 2019), attachment issues (Happé et al., 2017), and in the establishment and recognition of group affiliations (Verosky et al., 2013). Impairments in facial recognition, potentially the primary way in which we recognise others (Bruce & Young, 1986), are also thought to contribute to the symptoms observed in autism spectrum disorder (Boucher & Lewis, 1992; Wallace et al., 2008, Hedley et al. 2011).

Within the general population (i.e., those without a clinical diagnosis), individuals at the extremes of face recognition ability are labelled as either ‘prosopagnosic’ (if their face recognition is very poor) or as a ‘super recogniser’ (SR; if they exhibit superior face recognition). The majority of research interest thus far has focussed on prosopagnosic individuals (e.g., Duchaine & Nakayama, 2005; Duchaine & Nakayama, 2006b; Susilo & Duchaine, 2013; Bate & Tree, 2017; Towler et al., 2017; see Corrow et al., 2016; Cook & Biotti, 2016; Geskin & Behrmann, 2018 for recent reviews), particularly those who have suffered from face recognition impairments throughout life rather than as a result of brain trauma (developmental prosopagnosics; DPs). However, more recent work with SRs has probed the specificity and nature of their enhanced face recognition ability (e.g., Bate, Bennetts, et al., 2019a; Bate, Frowd, et al., 2019b; Bobak et al., 2016a; Russell et al., 2009).

A single test that is sensitive to individual differences across the full range of facial recognition performance is hard to design—tests designed to identify DPs are likely insensitive to the range of ability needed to distinguish SRs from those with good recognition within the normal range, and the converse is true for tests designed to identify SRs. This paper therefore presents the development and validation of a test that is able to measure individual differences in the typical range of performance, as well as in the range characterised by DPs and SRs.

A test suitable for the full range of performance should clearly consist of items of varying degrees of difficulty: with easier items allowing those with DP to be distinguished from those at the lower end of typical performance, and more challenging items allowing SRs to be distinguished from those at the higher end of typical performance, and with individual differences in ability within DPs and SRs also detectable. Establishing the difficulty of items in a task that can be completed successfully using a variety of strategies is not straightforward, however. Difficulty could be established by administering items to a large sample of participants and recording average performance. If performance is taken as reflecting difficulty, then items can be selected that span a wide range of difficulty. If there are reliable differences between the strategies or processing styles of different groups, however, (for example if typical observers process faces holistically while those with developmental prosopagnosia or Autism Spectrum Disorder process faces in a piecemeal fashion; Gauthier et al., 2009; Joseph & Tanaka, 2003; Avidan et al., 2011; DeGutis et al., 2012; Palermo et al., 2011; though see Biotti et al., 2017; Brewer et al., 2019; Faja et al., 2009), the items may be poorly calibrated for those populations with atypical strategies, with relative difficulty of items varying as a function of observer group. The sensitivity of such tests would therefore be biased towards the typical population. Items calibrated based on performance of atypical groups would suffer the same limitation when used with typical samples.

To overcome this problem, the current test of face processing determined item difficulty in a non-biased, objective^1^ manner, using the performance of facial recognition algorithms. Despite ethical concerns concerning the applied use of such algorithms (Mazura et al., 2012; Klare et al., 2012), and findings suggesting that performance of some algorithms may be affected by ethnicity in the same way as seen in some human recognisers (e.g., Irhebhude et al., 2019), it is clear that, on average, performance of the leading face recognition algorithms is at least equal to, and regularly better than, that of human recognisers (Phillips et al., 2018; Phillips & O'Toole, 2014). Note that several of the leading algorithms are based on deep neural networks, meaning that they are ‘black boxes’ in which the strategies/rules determining performance are difficult to determine. Importantly, however, it is not essential that such algorithms use similar strategies to human observers; indeed, if there are reliable differences in the strategies used by different human sub-populations in recognising faces, then it is preferable that algorithms do not utilise the same strategy as (typical) human observers—all that is required is that identity recognition is accurate. Accordingly, three leading facial recognition algorithms (FaceSoft, Azure Face Recognition, and AWS Rekognize) were used to determine the similarity of pairs of face images. Pairs of faces were selected as potential stimuli if similarity judgments were consistent across algorithms.

These face pair stimuli, and their objective degree of similarity, were used to derive a stimulus set that varied in difficulty when used in a face matching task requiring participants to determine whether two images of a face were of the same individual, or different individuals. Similarity is therefore related to difficulty in the following manner: very similar face images of the same person, and very different images of different people, are likely to be easier to judge correctly. In contrast, very different images of the same person, and very similar images of different people, are likely to be more difficult to judge correctly. Thus, sorting face pairs of the same individual and face pairs of different individuals by objective similarity allows the creation of items of increasing difficulty when used in a matching test. Note that such a test measures what has been referred to as face perception or face matching—determining whether instances of a face are exemplars of the same facial identity—not what has been referred to as face memory or identity recognition (labelling a face as a particular identity). Thus, it is possible for individuals to perform well on such a test, to be able to determine that Individual A is not Individual B, but not know that individual A is Derek Trotter and Individual B is Rodney Trotter. Face matching is necessary for identity recognition, however, as one must be able to determine that a particular face stimulus is a token of type ‘Derek Trotter’s face’, in order to be able to recognise Derek Trotter (Bruce & Young, 1986).

Across five studies, we report the development of the task (named the Oxford Face Matching Test; OFMT), the range of performance across individuals, the relationship between performance on the OFMT and standard face recognition tasks, the test-retest reliability of the OFMT in comparison to standard measures, and validation of the task in a group of DPs and SRs. These final studies allowed perception- and memory-based accounts of the atypical face recognition in DPs and SRs to be tested.

## Study 1

Study 1 aimed to develop the OFMT and to establish the range of performance on the measure to assess its usefulness as a measure of individual differences. In addition, scores on the OFMT were compared to a standard measure of face processing designed to identify DPs, the Cambridge Face Memory Test (CFMT; Duchaine & Nakayama, 2006a), and a self-report measure of face recognition ability, the 20-Item Prosopagnosia Index (PI-20; Shah et al., 2015, Gray et al., 2017). Participants completed both the long-form of the OFMT (which takes approximately 20 minutes), and a short form version designed for testing situations requiring an accelerated procedure (which ranges from 4 to 10 minutes in length).

## Methods

### Selection of stimuli

Pairs of face stimuli were selected from databases, both publicly available (the FERET dataset provided by DARPA; Phillips et al., 1997; Phillips et al., 1998) and databases held by the authors. All databases contained ground truth data as to whether the face pairs were images of the same person (‘same pairs’), or of different people (‘different pairs’). Images were naturalistic and varied in age and gender, but selected images were cropped to a 3:4 ratio to ensure that the face occupied most of the presented stimulus. Images of participants wearing glasses were excluded. All images were of Caucasian people, frontal, presented with hair, but without background context. Images were both assessed by face recognition algorithms and presented to participants in greyscale, with no other low-level processing conducted.

Over three million face pairs were selected for similarity assessment by three algorithms: AWS Rekognition (https://aws.amazon.com/rekognition/), FaceSoft (retrieved from http://facesoft.io/) and Azure Face Recognition (https://azure.microsoft.com/en-us/services/cognitive-services/face/). The algorithms provide a similarity index ranging from 0 to 1 (or 0 to 100, scaled to 0 to 1 for ease of comparison). Face pairs were placed into 20 similarity ‘bins’ based on their similarity score, each 0.05 units wide (spanning the similarity scores from 0 to 1). Any face pair for which there was a difference of two or more bins on the similarity judgment between algorithms was excluded. For the remaining pairs, a mean similarity index was calculated across the three algorithms, and pairs were then assigned to one of the 20 bins based on their average similarity index. Ten image pairs were randomly selected from each bin for use as stimuli in the OFMT, with the constraint that there were equal numbers of same and different pairs (resulting in a total of 200 stimulus pairs, 100 depicting the same identity and 100 depicting two different identities).

Stimulus similarity bins were then used to construct a range of difficulty bins. 5 same pairs and 5 different pairs from each of the 20 similarity bins were allocated to 20 new bins, arranged in order of ‘difficulty’, such that bin 1 contained the most similar same pairs and most dissimilar different pairs, while bin 20 contained the most different same pairs and most similar different pairs.

### Tasks

#### OFMT – long form

**Fig. 1:**
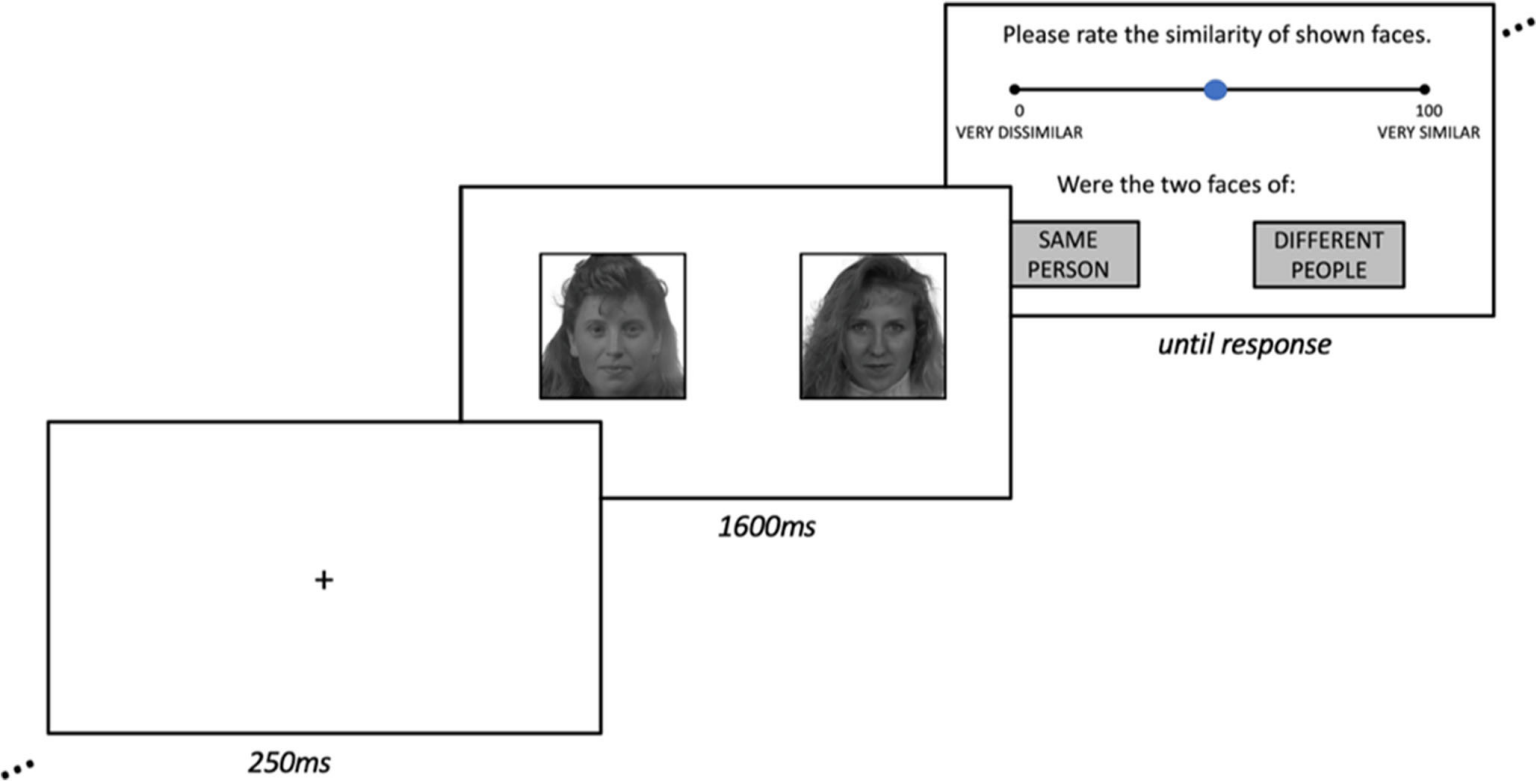
Sample trial from the Oxford Face Matching Test – long form. Participants were shown two faces concurrently for 1600 ms. They were asked to judge the similarity of the faces using a slider ranging from 1 (very dissimilar) to 100 (very similar), and to judge whether the face images were of the same person, or different people. Note: relative text size on the final frame enlarged to aid legibility

Participants were shown pairs of faces side-by-side for 1600 ms and asked to make a judgment about the similarity of the faces on a scale of 1 (very dissimilar) to 100 (very similar), as well as to judge whether the face images were of the same person, or different people (see Fig. 1). A standard presentation time was used (rather than trials proceeding upon the participant’s response) due to a concern that individuals with prosopagnosia may assume that they will perform poorly, and thus respond quickly without giving stimuli due consideration, in order to finish quickly, or may compensate for their difficulties by studying the faces for longer time periods. Pilot testing with input from prosopagnosic individuals revealed that 1600 ms was deemed sufficient time for judgements to be made as to the degree of similarity, and whether face images were of the same or different individuals. All 200 stimulus pairs were presented in a random order.

#### Attention check trials

The long-form version of the OFMT included an additional 10 attention check trials. These trials were designed to be easy even for those with severe face processing impairments. Five same pairs were constructed using exactly the same images of a face, while five different pairs were constructed using faces of different genders. Note that pairs of faces of different genders consistently yielded similarity indices near zero, which is why they were selected for attention check trials. There were no mixed-gender trials in the OFMT other than for these attention check trials. Any participant making two or more mistakes on these trials was excluded prior to analysis.

#### OFMT – short form

Trial structure was as detailed above for the long form of the task except that participants were not required to make a similarity judgement. Rather than viewing all 200 face pairs, participants completed a reduced number of trials according to the logic of a staircase procedure, whereby difficulty increased following good performance, and decreased following poor performance.

Participants initially completed 50 trials of medium difficulty (10 trials from each bin in bins 8–12, in a randomised order). Based on their performance, three outcomes were possible: (1) Participants who did not achieve 80% accuracy on at least two consecutive bins (e.g., bins 8 and 9, bins 9 and 10, etc.) were presented with easier trials from consecutively lower bins until they achieved 80% accuracy on two consecutive bins or no more trials remained (i.e., they failed to achieve 80% accuracy on bins 1 and 2). (2) Participants who achieved 80% accuracy on the most difficult two bins (11 and 12) were presented with more difficult trials from consecutively harder bins until two consecutive failures to reach 80% accuracy per bin were observed, or no more trials remained (because participants achieved at least 80% accuracy on bins 19 and 20). (3) Participants who achieved 80% accuracy for any two consecutive bins (other than bins 11 and 12) completed no more trials. Participants always saw all trials from a bin before finishing the task or proceeding to the next bin. The threshold was determined to be the average of the two highest consecutive bins completed with 80% accuracy. Note that an 80% accuracy threshold was used, as this represents performance significantly above chance level (with an alpha of < .05).

#### Cambridge Face Memory Task (CFMT; Duchaine & Nakayama, 2006a)

The CFMT has been used extensively to measure face recognition, and was designed to measure unfamiliar face memory skills to distinguish those with prosopagnosia from typical perceivers. Participants learn six target faces at the beginning of the test, after which they are tested on three-alternative forced-choice trials. On each trial, two images are distractors and one is an image of a learned target identity. The test is divided into three stages of increasing difficulty, involving 18 test trials with no change of viewpoint or lighting, 30 trials with viewpoint and lighting changes, and 24 trials with viewpoint and lighting changes along with the addition of visual noise.

20-Item Prosopagnosia Index (PI-20; Gray et al., 2017; Shah et al., 2015)

The PI-20 is a self-report questionnaire used as a screening tool to identify people with difficulties in face recognition. It has previously been validated with the CFMT, and has been shown to be able to distinguish DPs from the neurotypical population (Gray et al., 2017). The survey consists of 20 items on which respondents can report face recognition difficulties in everyday life.

#### Autism Spectrum Quotient-50 (AQ-50; Baron-Cohen et al., 2001)

The AQ-50 is a self-report diagnostic questionnaire used to determine the severity of Autism Spectrum Disorder symptoms in adults with normal intelligence (Woodbury-Smith et al., 2005).

### Participants

Across all studies, any participants who met one or more of the following criteria were excluded: technical difficulties prevented completion of the task; did not meet recruitment criteria (over 18 years old, no current or past psychiatric or other neurodevelopmental diagnoses, no current use of psychotropic medications); failed to attend to the task (determined by performance on attention check trials). Additionally, unless recruited as part of a DP group, participants were excluded if they met any of the following criteria: scored more than 2 standard deviations below the group mean on the CFMT test (used to confirm prosopagnosia, Duchaine & Nakayama, 2006a); their PI-20 scores indicated the possibility of prosopagnosia (Shah et al., 2015; Gray et al., 2017). Finally, any participant was excluded if their Autism Spectrum Quotient-50 questionnaire scores indicated high autistic traits (scores of 32 or higher; Baron-Cohen et al., 2001).

For Study 1, 45 participants with normal or corrected-to-normal vision participated in the study in the laboratory. Four participants were excluded for failing the attention checks, producing a final sample of 41 participants (24 female, mean age 26.6 years, SD = 3.9). Participants completed the PI-20, CFMT, and the OFMT long and short forms. The OFMT short form was always completed first as it was deemed to be most susceptible to interference from prior completion of the other tasks, and the order of the other tasks was randomised for each participant. All tasks were completed using the Gorilla Experiment Builder platform (www.gorilla.sc).

### Results and discussion

**Fig. 2:**
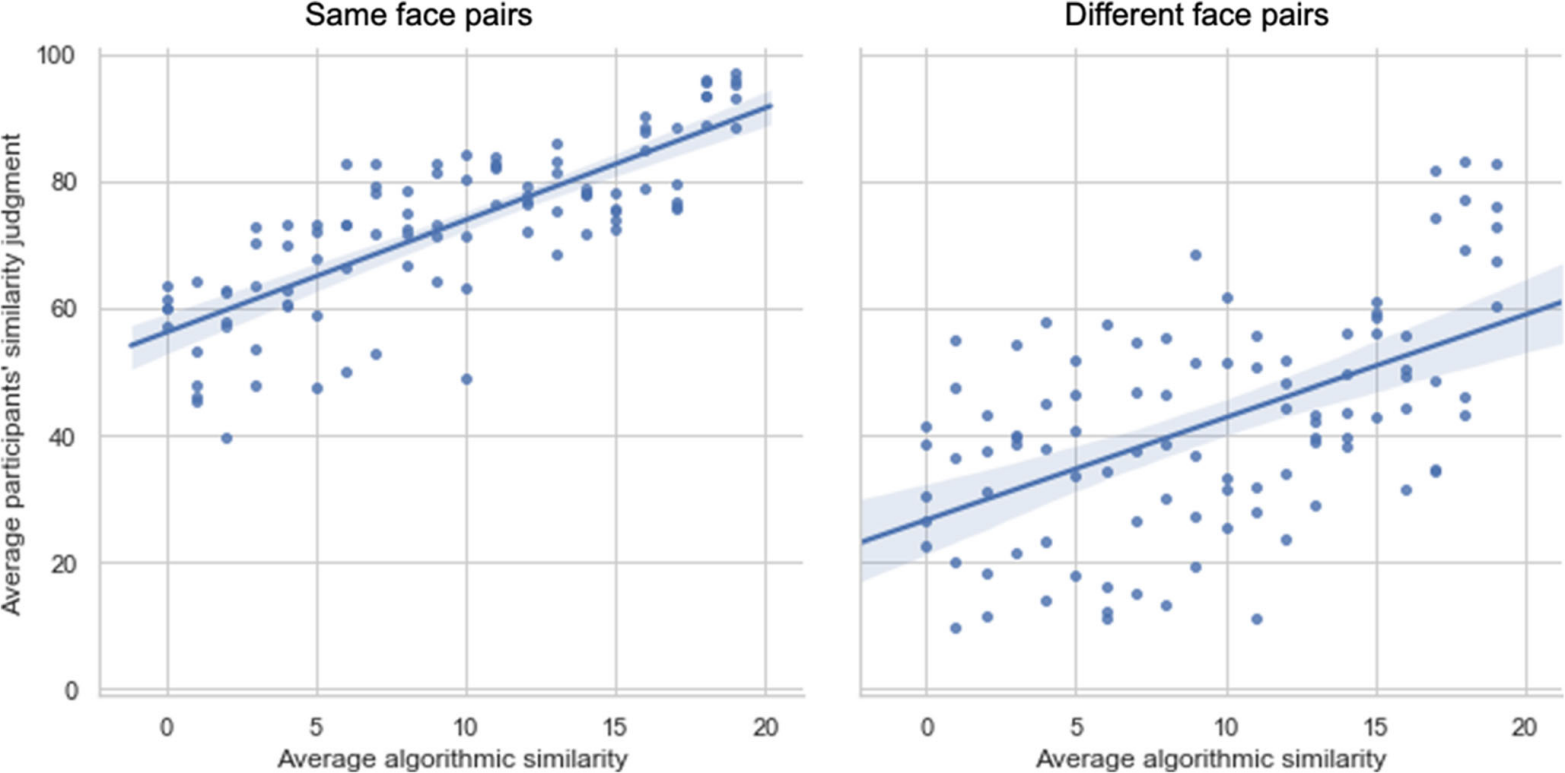
Relationship between participants’ similarity judgments and average algorithmic similarity values (expressed here in bins of 1–20). Each point shown on the graphs represents average group judgments about the similarity of a given face pair (5 same pairs and 5 different pairs are shown for each difficulty level)

Accuracy on the OFMT long form ranged between 59.5% and 84% (raw scores: 119–168), with a mean of 74.1% (SD = 5.4%, raw scores: 148.2 ± 10.8). A small-to-moderate relationship was observed between accuracy on the OFMT and CFMT (*r*(39) = .32, *p* < .05). A moderate relationship was observed between average algorithmic similarity values and judged similarity for same-face pairs (*r*(39) = .42, *p* < .05) and a small-to-moderate relationship was for different-face pairs (*r*(39) = .32, *p* < .05; Fig. 2). There was a small, non-significant relationship between PI-20 scores and performance on the long form of the OFMT (*r*(39) = −0.14, *p =* .38).

**Fig. 3:**
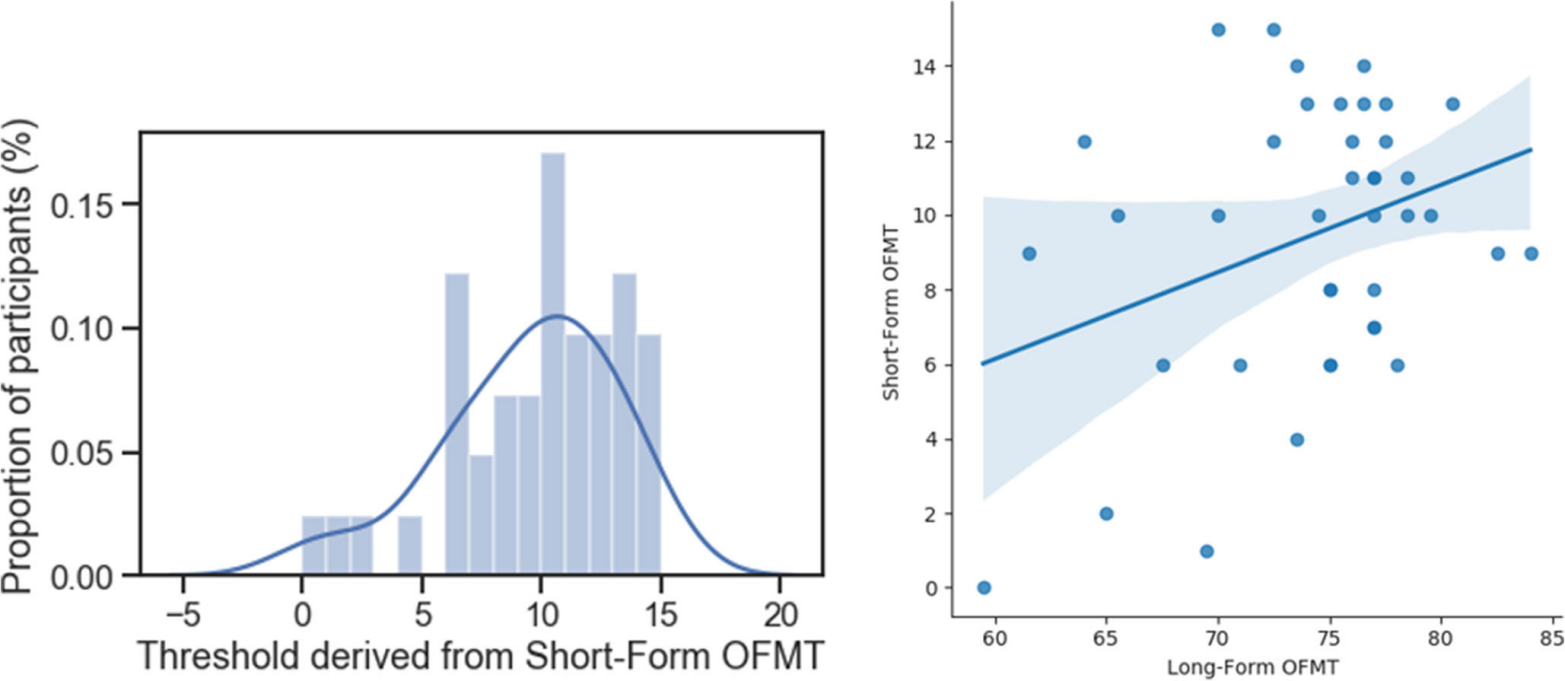
Distribution of thresholds derived from the short form of the Oxford Face Matching Test (OFMT; left panel). The right panel depicts the relationship between accuracy on the long form of the OFMT (200 trials) and the thresholds derived from the short form (between 50 and 110 trials, depending on performance). The shaded area in the right panel indicates the 95% confidence interval estimated using a bootstrapping method with replacement

A wide range of thresholds was observed using the short form of the OFMT (Fig. 3, left panel). For participants for whom the threshold was found (one participant did not complete the task with >80% accuracy in bins 1 and 2), thresholds ranged from 1 to 15 (M = 9.7, SD = 3.4). In addition, a small-to-moderate statistically significant relationship (*r*(39) = .34, *p* < .05) was observed between the threshold derived for each participant from the short form of the OFMT and accuracy derived from the long form of the OFMT (Fig. 3, right panel).

Data from Study 1 indicate that both the long and short forms of the OFMT can be completed and understood by participants and produce a wide range of performance. Performance on the OFMT long form correlated significantly, but only moderately, with performance on the CFMT. This is to be expected as the difficulty of trials in the CFMT is increased, in part, through the addition of visual noise. This manipulation undoubtedly increases the difficulty of the task but may mean that individual differences in part reflect the ability to cope with visual noise, rather than pure face perception (it should be noted, however, that performance on the CFMT items with visual noise is significantly correlated with performance on items without visual noise; Duchaine & Nakayama, 2006a). Perhaps more importantly, it is also the case that performance on the CFMT is in part determined by individual differences in face learning/face memory, in contrast to the OFMT in which performance is determined by face perception. Data also suggest that the short form of the OFMT is, in its current form, a noisy proxy for the long-form test. The small-to-moderate correlation between the two forms of the OFMT is likely a result of the reduced number of items in the short form, leading to a noisy estimate of an individual’s ability.

## Study 2

Study 2 constituted a direct replication of Study 1, except that participants completed the study online rather than in the laboratory.

### Methods

#### Participants

One hundred and six participants with normal or corrected-to-normal vision participated in the study online for payment. Nine participants were excluded for failing the attention check and/or failing to finish the task, after which the sample contained 97 participants (49 female, mean age 27.2 years, SD = 7.71).

#### Procedure

The procedure for Study 2 was exactly the same as for Study 1, except that participants completed the tasks online, via the Prolific.co recruitment platform.

#### Results

**Fig. 4:**
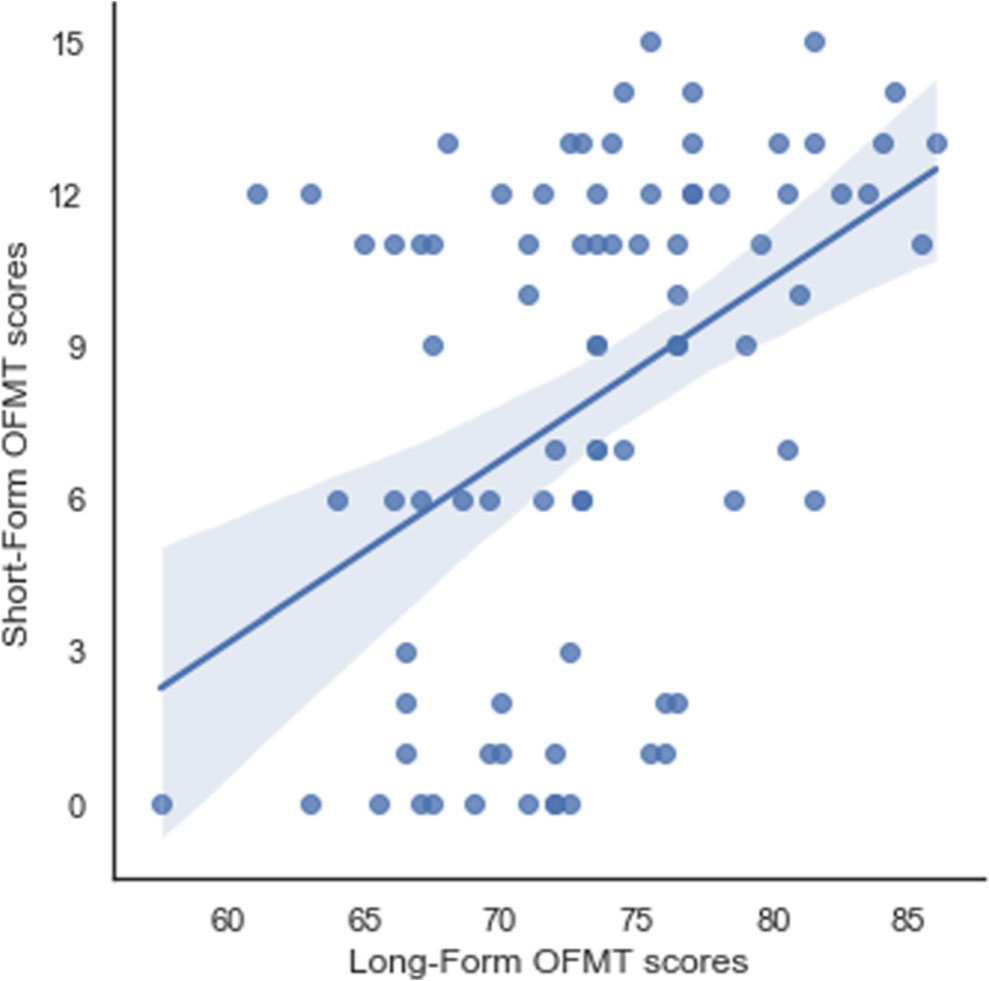
The relationship between accuracy on the long form of the OFMT (200 trials) and the thresholds derived from the short form (between 50 and 100 trials, depending on performance)

Accuracy on the OFMT long form ranged from 52% to 86% (raw scores: 104–186), with a mean performance of 72.9% (SD = 5.7%, raw scores: 145.8 ± 11.4). As in Study 1, a small-to-moderate statistically significant correlation was observed between accuracy on the OFMT long form and CFMT scores (*r*(95) = .34, *p* < .05). A moderate correlation of *r*(95) = .44 (*p* < .05) was observed between the short-form thresholds and long-form accuracy (Fig. 4). There was also a small relationship between PI-20 scores and performance on the long form of the OFMT, which approached significance (*r*(95) = −.20, *p* = .06).

**Fig. 5:**
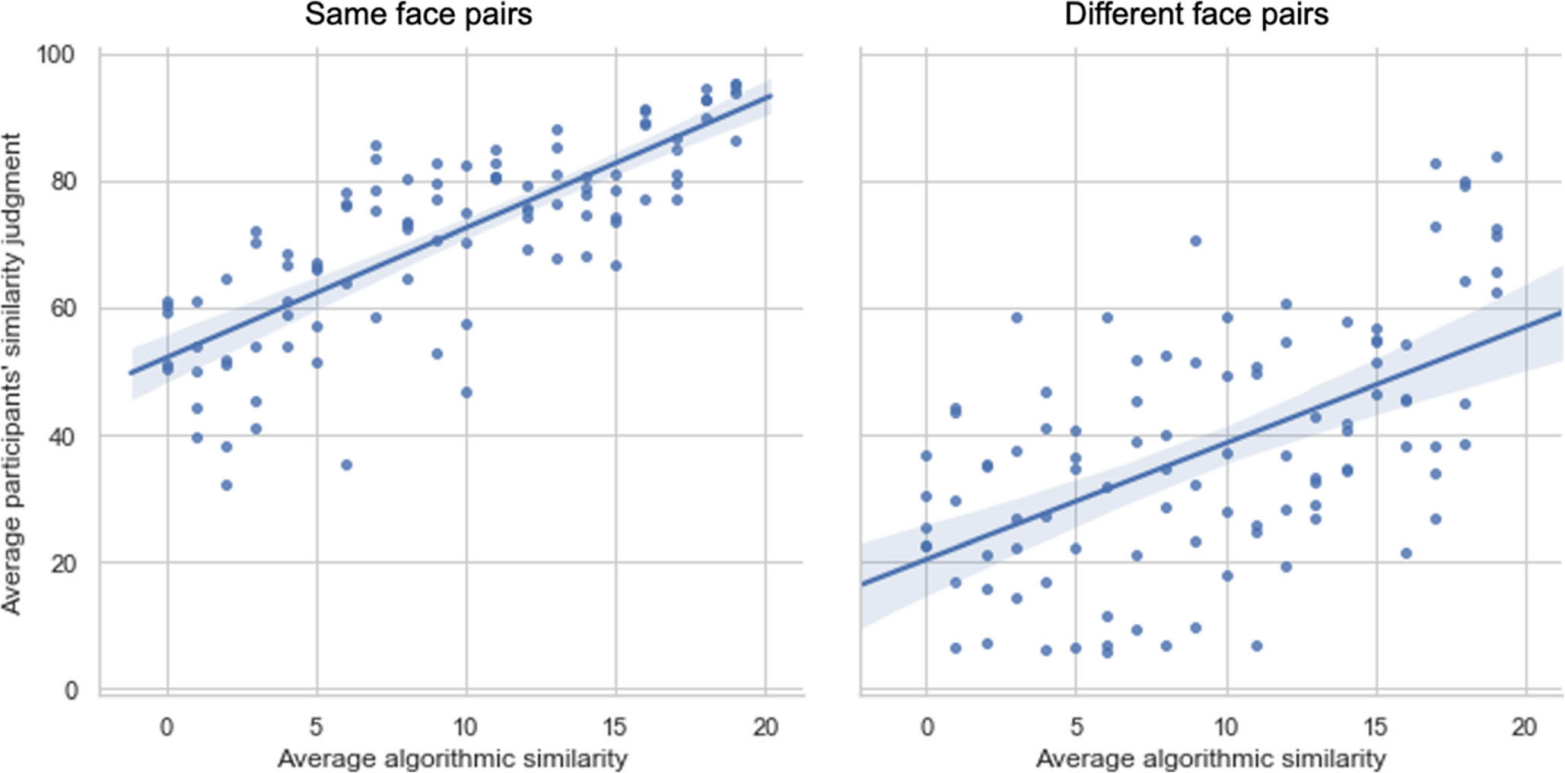
Relationship between participants’ similarity judgments and average algorithmic similarity values (expressed here in bins of 1–20) for same-face (left panel) and different-face (right panel) trials

In line with the findings of Study 1, a moderate relationship between algorithmic similarity and judged similarity was observed for same-face pairs (*r*(95) = .44, *p* < .05) and a small-to-moderate relationship was observed for different-face pairs (*r*(95) = .34, *p* < .05) (Fig. 5).

#### Analyses across Study 1 and Study 2

Given that the design of Study 1 (conducted in-person) and Study 2 (conducted online) was identical, the following analyses were conducted on data from both studies.

**Fig. 6:**
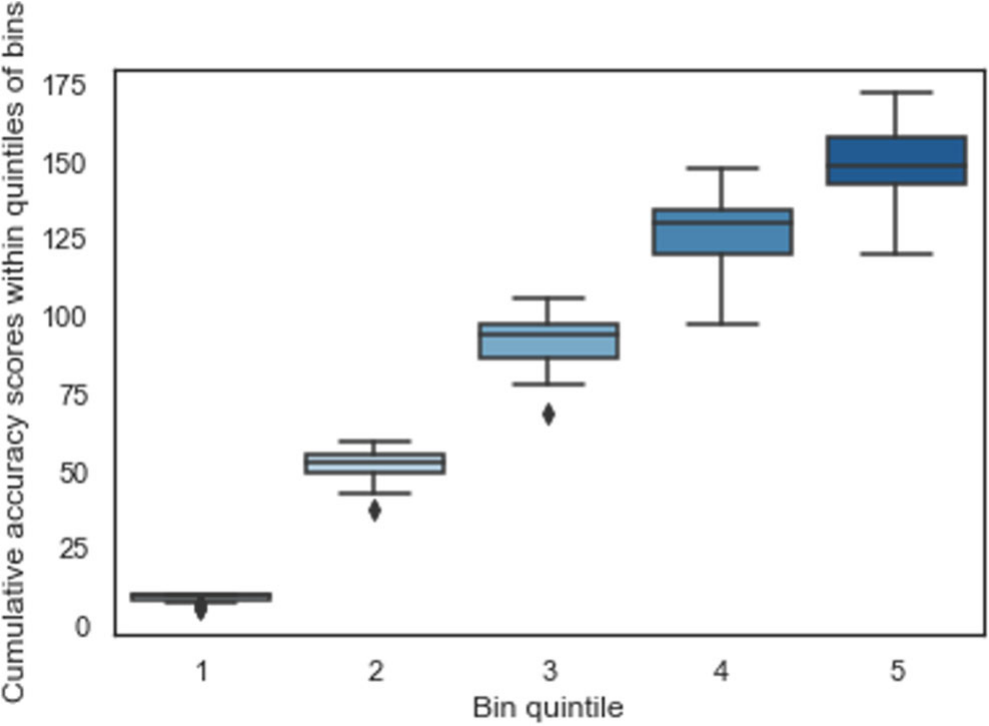
The increasing variance of neurotypical performance is shown as function of an increase in difficulty (indicated by bin quintile) across the test. The boxes represent the **inter-quartile range of cumulative scores across participants for each bin quintile**, the lines within boxes show median performance, and whisker-lines represent the extremes of the distribution with outliers indicated by diamonds

The logic of the OFMT rests on the assumption that similarity will be related to difficulty, such that as objective similarity of face images increases, the difficulty of different pairs increases, and the difficulty of same pairs decreases. The 20 difficulty bins were constructed so that bin 1 contains the most similar same-identity pairs and most dissimilar different-identity pairs, while bin 20 contains the most dissimilar same pairs and the most similar different pairs. Thus, the trials in bin 1 are hypothesised to be the easiest and the trials in bin 20 the most difficult. Evidence for the hypothesised relationship between similarity and difficulty was obtained by regressing bin number against performance on the data from Studies 1 and 2. This analysis revealed that bin was a significant predictor of performance (*F*(1, 18) = 95.99, *p* < .001, *R*^2^ = 0.842). Performance ranged from a mean of 91.2% (SD = 10.3%) at bin 1, to a mean of 38.6% (SD = 14.5%) at bin 20. To confirm the validity of the more difficult bins, we investigated the hypothesis that the variance in accuracy across participants should increase as trial difficulty increases. We analysed the distribution of variance at each of the quintiles of difficulty shown in Fig. 6 and found that, as expected, Mauchly’s test of sphericity indicated unequal variances across quintiles (χ2(5) = 13.66, *p* < .05).

### Discussion

Study 2, a replication of Study 1 using online testing, replicated the results of Study 1. Both studies demonstrated a wide range of performance on both the long and short forms of the OFMT, and that although the short form correlated significantly with the long form, there was only a moderate correlation between the long and short forms (*r* = .42) when data were collapsed across Studies 1 and 2. Correlations between the OFMT and CFMT were small-to-moderate in both studies. When data across studies were combined, the hypothesised relationship between similarity of face pairs, as assessed objectively by algorithms, and item difficulty was supported by the performance data across bins. Additionally, the inclusion of more difficult items increased variance in scores as would be expected. These data all support the validity of the OFMT.

## Study 3

Study 3 assessed the test-retest reliability of the OFMT and compared it to three existing measures: the CFMT and PI-20, as widely used objective and subjective measures of face recognition, and the Glasgow Face Matching Task (GFMT; Burton et al., 2010). The GFMT was introduced into the testing battery as it is a test of face matching, like the OFMT, without the face memory requirement of the CFMT. To our knowledge, there are only three published studies that have assessed the test-retest reliability of the CFMT. The first tested a large sample (*n* = 389) of participants with a delay of 6 months and found a correlation of .70 between testing sessions for identical versions of the CFMT and .76 between the original CFMT and a version with computer-generated face stimuli (Wilmer et al., 2010). The second study used an Australian version of the CFMT (a different stimulus set to the original version of the test) and found a correlation of .92 with a 24-hour delay between test sessions (McKone et al., 2011). Finally, a recently published paper found a test-retest reliability of .68 in a group of 70 developmental prosopagnosics tested in the laboratory and online (Murray & Bate, 2020).

### Methods

#### Participants

Seventy-two participants with normal or corrected-to-normal vision participated in the study online. One was excluded for failing the attention check. Data for 71 participants are reported (43 female, mean age 31.6, SD = 7.8 years).

#### Procedure and tasks

Participants completed the PI-20, CFMT, GFMT, and the long-form version of the OFMT online via the Prolific recruitment platform. Participants completed all tasks twice, with 14 days between each completion. Task order was randomised at each testing time.

#### Glasgow Face Matching Task (Burton et al., 2010)

The GFMT is a standard test used to assess unfamiliar face matching ability. Participants are shown two faces of either the same individual (match trials) or different individuals (mismatch trials) for an unlimited amount of time and asked to determine if faces belong to the same person or different people. The short form used here consists of 20 match and 20 mismatch trials, presented in a random order.

#### Results and discussion

Test-retest reliability ranged between acceptable and good for all measures: Cambridge Face Memory Test (*r*(69) = .67, *p* < .05), Glasgow Face Matching Task (*r*(69) = .77, *p* < .05), Oxford Face Matching Test (*r*(69) = .75, *p* < .05), and the 20-Item Prosopagnosia Index (*r*(69) = .89, *p* < .05). Across the three objective measures (OFMT, CFMT, and GFMT) test-retest reliability did not significantly differ (GFMT:OFMT: *z* = −0.375, *p* > .05; CFMT:GFMT: *z* = −0.121, *p* > .05; CFMT:OFMT: *z* = −0.509, *p* > .05). Test-retest reliability of the OFMT is therefore as high as the reliability of existing face processing measures.

A significant change in scores between testing days was not observed for the CFMT (T1: *M* = 56.7, *SD* = 10.2, T2: *M* = 55.5, *SD* = 10.6, *t*(70) = 1.16, *p* = .25), GFMT (T1: *M* = 32.5 (81.3%), *SD* = 4.5 (11.25%), T2: *M* = 32.7 (81.8%), *SD* = 5.3 (13.3%), *t*(70) = −0.50, *p* = .62) or the PI-20 (T1: *M* = 44.8, *SD* = 11.3, T2: *M* = 44.5, *SD* = 11.9, *t*(70) = 0.41, *p* = .68). A small but significant increase in average scores was observed for the OFMT (T1: *M* = 152 (76.0%), *SD* = 10.6 (5.3%), T2: *M* = 155.2 (77.6%), *SD* = 12.8 (6.4%), *t*(70) = −3.1, *p* < .05).

Further, results from Study 3 corroborate findings reported in Studies 1 and 2. There was a small-to-moderate relationship between the scores on the CFMT and OFMT at time 1 of testing *r*(69) = .41, *p* < .01) as well as time 2 *(r*(69) = .38, *p* < .01). A stronger relationship, as expected, was observed between OFMT and GFMT, the two matching tasks, both at time 1 of testing (*r*(69) = .46, *p* < .01) and time 2 of testing (*r*(69) = .59, *p* < .01). A small, non-significant, relationship was observed between PI-20 and OFMT performance at time 1 (*r*(69) = −.22, *p* = .07) and time 2 *(r*(69) = −.20, *p* = .11). Relationships between all measures are shown in Table 1. Finally, a small-to-moderate relationship was observed between average algorithmically derived similarity values and participants’ judgements for different-face pairs for both time points (time 1: *r*(69) = .29, *p* < .05, time 2: *r*(69) = .32, *p* < .05) and a moderate relationship for same-face pairs for both days (time 1: *r*(69) = .49, *p* < .05; time 2: *r*(69) = .47, *p* < .05).

**Table 1:** Relationships between all face processing measures included in Study 3

Variable	CFMT (T1)	CFMT (T2)	PI-20 (T1)	PI-20 (T2)	GFMT (T1)	GFMT (T2)	OFMT (T1)	OFMT (T2)
**CFMT (T1)**	-							
**CFMT (T2)**	.67^**^	-						
**PI-20 (T1)**	−.22	−.22	-					
**PI-20 (T2)**	−.16	−.16	.89^**^	-				
**GFMT (T1)**	.56^**^	.38^**^	−.25^*^	−.18	-			
**GFMT (t2)**	.58^**^	.39^**^	−.30^*^	−.27^*^	.77^**^	-		
**OFMT (T1)**	.41^**^	.41^**^	−.22	−.25^*^	.46^**^	.41^**^	-	
**OFMT (T2)**	.42^**^	.38^**^	−.18	−.20	.45^**^	.59^**^	.75^**^	-

Correlations significant at the 0.01 level are denoted with two asterisks (**) and correlations significant at the 0.05 level are denoted with a single asterisk (*). Testing time 1 is denoted by T1 and testing time 2 by T2. Testing times were 14 days apart. Given test-retest reliability data (reported in Study 3), correlations between all measures are high, except for correlations between the self-report PI-20 and the objective measures (OFMT, GFMT, and CFMT)

The results of Study 3 suggested that the test-retest reliability of the OFMT long form was at least as good as that of the CFMT and GFMT. All tests had acceptable to good reliability, indicating their suitability as measures of individual differences in face processing (under the assumption that face processing ability is a trait that shows little variation across time). With test reliability established, Study 4 again aimed to test the validity of the OFMT.

## Study 4

Study 4 had two aims, with the first related to test validation across the full range of performance. The idea behind the OFMT is that a single test should be sensitive to both individual differences in face perception in the typical range, and individual differences in both the DP and SR range of performance. As such, Study 4 tested the performance of a group of DPs and a matched, neurotypical control group on the OFMT and other standard tests of face processing (CFMT and GFMT).

The second aim of Study 4 was to distinguish between two different forms of face processing deficit in DP, namely a perceptual deficit and a memory deficit. Briefly, there has been a debate in the literature between accounts suggesting that most individuals with DP are able to form an intact perceptual representation of faces but have difficulty learning and/or remembering facial identities (the ‘memory hypothesis’, Jackson et al., 2017; Stollhoff et al., 2011), and accounts arguing that many individuals with DP have difficulties forming perceptual representations of faces in addition to, or instead of, face memory deficits (the ‘perceptual hypothesis’; see Biotti et al., 2019; Dalrymple et al., 2014b), raising the possibility of multiple subtypes of DPs characterised by different types of impairments.

The inclusion of both the OFMT and CFMT allows the perceptual and memory hypotheses to be tested, due to the fact that performance on the OFMT is governed by face perception with minimal face memory demands (simultaneous presentation of the two face stimuli minimises the need to hold stimuli in memory), while the CFMT requires face perception and face memory. Thus, if face perception only is impaired in DP then one would expect any impairment in performance on the CFMT in DPs to be fully explained by performance on the OFMT. If only face memory is impaired in DP then one would expect DP to produce a selective deficit on the CFMT and not OFMT, while if both face perception and face memory are impaired then one would expect deficits on both tasks, but that the deficit on the CFMT would remain after performance on the OFMT is accounted for.

### Methods

#### Participants

Thirty-one developmental prosopagnosic participants with normal or corrected-to-normal vision participated in the study online. Participants were selected as DPs from a pre-existing database of clinically assessed participants with face perception difficulties. They met the criteria for impaired performance (defined as 2 SDs below the mean) on both the CFMT and the Famous Faces Test (following primary criteria outlined in Barton & Corrow, 2016). Two of the participants did not show impaired performance on the Famous Faces Test, but their PI-20 scores (79 and 88, respectively) indicated a high degree of issues with face recognition in everyday life. As these participants were not outliers on any measures, they were included in the analysis. All DP participants scored higher than 70 on the PI-20. One prosopagnosic participant was excluded for failing to finish all measures, so a sample of 30 DPs is reported (23 female, mean age 42.6, SD = 10.7). An age- and gender-matched sample of 30 neurotypical participants (23 female, mean age 41.8, SD = 4.1) was recruited as a control group (age: *t*(58) = .89, *p* = 0.38; gender: *Χ* ^*2*^(1) = .66, *p* = .80). No neurotypical participants were excluded for failing the attention check trials.

### Procedure

Participants completed the PI-20, CFMT, GFMT, and long-form version of the OFMT in a randomised order.

### Results and discussion

**Fig. 7:**
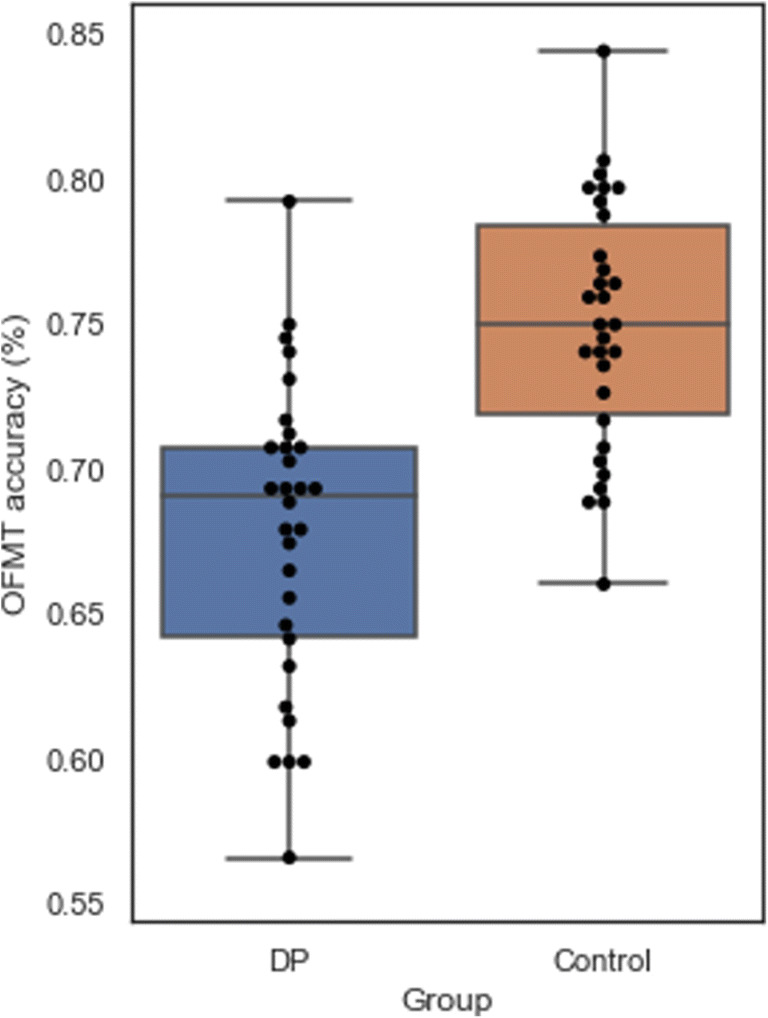
Performance of a group of individuals with developmental prosopagnosia and an age- and gender-matched control group on the Oxford Face Matching Test. The boxes represent the inter-quartile range, the lines within boxes show median performance, and whisker-lines represent the extremes of the distribution (excluding outliers)

While the neurotypical control group’s mean accuracy on the OFMT was 75% (SD = 4.31%, range 66.0% to 84.5%, raw scores: 132–170, 150 ± 8.62), the DP group scored significantly worse, with a mean accuracy of 67.8% (SD = 5.28%, range 56.5% to 79.0%, raw scores: 113–158, 135.6 ± 10.56; *t*(58) = 5.78, *p* < .001; Fig. 7). As expected (given all DP participants were classified as DP based in part on CFMT performance in a prior testing session), the DP group (mean = 34.6, SD = 5.6) performed significantly worse on the CFMT than the control group (mean = 53.3, SD = 10.9), *t*(58) = 8.81, *p* < .001. There was also a significant difference in self-reported difficulties with face perception between the DP group (mean = 84.07, SD = 6.0) and the control group (mean = 45.60, SD = 9.81, *t*(58) = 18.33, *p* < .001) as measured with the PI-20. Finally, a significant difference was also observed in performance on the GFMT between the DP group (mean = 26.9, SD = 5.0, percentage scores: 67.3% ± 12.5%) and the control group (mean = 32.87, SD = 4.39, percentage scores: 82.2% ± 11.0%, *t*(58) = 4.91, *p* < .001). Relationships between all face-processing measures are shown in Table 2.

**Table 2:** Relationship between all face measures included in Study 4 across all participants. Two asterisks (**) denote a relationship significant at *p* < .01 level

	*n*	*M*	*SD*	Cambridge Face Memory Test (CFMT)	20-Item Prosopagnosia Index (PI-20)	Glasgow Face Matching Task (GFMT)	Oxford Face Matching Test (OFMT)
CFMT	60	44.4	13.2	-			
PI-20	60	64.8	21.0	−.77^**^	-		
GFMT	60	29.9	5.6	.66^**^	−.58^**^	-	
OFMT	60	142.8	12.0	.73^**^	−.63^**^	.75^**^	-

Within the DP group, a moderate correlation was observed between CFMT scores (note the restricted range due to inclusion of participants only if they meet criteria for DP diagnosis) and their overall accuracy on the OFMT (*r*(28) = .52, *p* < .05). The same correlation in the control group was also significant (*r*(28) = .59, *p* < .05; whole sample *r*(58) = .73, *p* < .001). The presence of a correlation between these two tasks in the DP group indicates that both tasks are sensitive to individual differences in the DP range of performance.

To address the perceptual and memory hypotheses, hierarchical multiple regression was used whereby OFMT scores were entered into step 1 of a model predicting CFMT scores, and then group (DP vs control) entered into step 2. The change in *R*^2^ from step 1 to step 2 (.16) was significant (*F*(1,57) = 28.94, *p* < .001), meaning that variance in CFMT scores could be accounted for by group over and above that explained by OFMT scores.

Results from Study 4 supported the validity of the OFMT. A group of individuals independently confirmed as DPs were significantly worse at the test than a matched neurotypical control group. Furthermore, OFMT scores correlated well with CFMT scores used, in part, to ‘diagnose’ individuals with DP. With respect to the debate concerning the perceptual and memory hypotheses of prosopagnosia, results supported both hypotheses. The DP group were significantly worse than the control group on the OFMT, a task with perceptual but not memory demands. The DP group were also significantly worse than the control group on the CFMT, a test with perceptual and memory demands, and this deficit in performance could not be fully accounted for by perceptual problems identified with the OFMT (as demonstrated using the hierarchical multiple regression analysis). Results therefore suggest that, as a group, individuals with DP have problems both with forming perceptual representations of faces and with learning/remembering facial identities. Indeed, when looking at individual cases, all but 2 of 30 DP participants scored below the control group’s median score on the OFMT, confirming that the vast majority of individuals with DP have perceptual difficulties with faces.

## Study 5

Study 5 was conceptually identical to Study 4, but instead examines the upper end of the face processing distribution. Study 5 attempted to validate the OFMT by examining the performance of a group of SRs, together with a matched neurotypical group, on the OFMT, CFMT, and GFMT. Study 5 also sought to ascertain whether the superior face recognition exhibited by SRs is a product of superior face perception, face memory or both, as previous studies have found mixed results (Bate, Frowd, et al., 2019b; Bobak et al., 2016b, c).

### Method

#### Participants

Thirty-two super recogniser participants (16 female, mean age 39.9, SD = 9.3) with normal or corrected-to-normal vision participated in the study online. They were recruited from a pre-existing database of super recognisers based on their superior performance on the CFMT+, a version of the CFMT designed to be more difficult than the standard version (Russell et al., 2009), using a cut-off score of 90 for inclusion as a SR (Bate et al., 2018; Bate, Frowd, et al., 2019b). An age- and gender-matched sample of 32 neurotypical participants (16 female, mean age 39.1, SD = 5.8) was recruited as a control group (age: *t*(62) = .36, *p* = 0.72; gender: *Χ* ^*2*^(1) = .62, *p* = .43). No participants were excluded from either sample for failing the attention check.

### Procedure

Participants completed the PI-20, CFMT+, GFMT, and the long-form version of the OFMT. In the CFMT+, as in the standard CFMT, participants learn faces of six individuals and are then tested on 72 trials in which they are required to select a single target face from a trio of faces. The CFMT+ extends the standard version of the CFMT by including an additional 30 more difficult trials to make the test suitable as diagnostic tool for use with SRs.

### Results and discussion

**Fig. 8:**
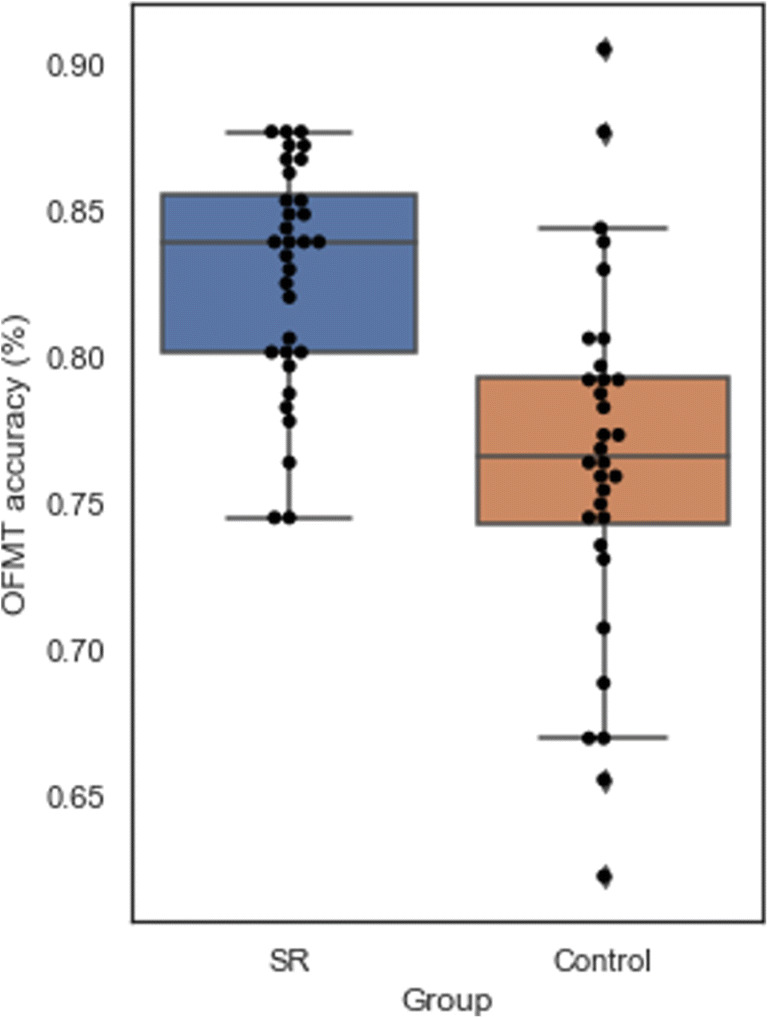
Accuracy scores of super recognisers and control participants on the Oxford Face Matching Test. The boxes represent the inter-quartile range, the lines within boxes show median performance, and whisker-lines represent the extremes of the distribution with outliers indicated by diamonds

While the control group’s mean accuracy on the OFMT was 76.5% (SD = 6.1%; range 62.5% to 90.5%, raw scores: 125–181, 153 ± 12.2), the SR group was significantly more accurate, with a mean accuracy of 82.8% (SD = 3.9%, range 74.5% to 88.0%, raw scores: 149–176, 165.6 ± 7.8; *t*(62) = 4.88, *p* < .005; Fig. 8). Twenty-nine out of 32 SRs scored above the control group’s mean performance on the OFMT, and 19 of 30 scored more than one standard deviation above the control mean. As expected (given SR participants were classified as SRs based on CFMT+ performance from a previous testing session), the SR group’s performance (mean = 67.3, SD = 3.0) was significantly better than the control group’s performance (mean = 54.6, SD = 12.1; *t*(62) = 5.77, *p* < .001) on the CFMT (note that while the SR status was confirmed on the basis of CFMT+ scores, CFMT scores are reported here). A significant difference in PI-20 scores was observed between SRs (mean = 28.4, SD = 4.0) and the control group (mean = 43.8, SD = 11.2, *t*(62) = 7.35, *p* < .001). Finally, a significant difference on the GFMT was also found between the control (mean = 32.8, SD = 5.6, percentage scores: 82.0% ± 14.0%) and SR (mean = 38.5, SD = 2.8, percentage scores: 96.3% ± 0.7%%, *t*(62) = 5.16, *p* < .005) groups.

Within the SR group, a significant correlation of moderate-large size was observed between CFMT+ accuracy (note the restricted range due to inclusion of participants only if they meet criteria for SR classification) and accuracy on the OFMT (*r*(30) = .50, *p* < .005), suggesting that these tasks are sensitive to individual differences in the high range of ability. The CFMT:OFMT correlation was of a similar size in the control group (*r*(30) = .52, *p* < .005; whole sample *r*(62) = .65, *p* < .001).

A number of super recognisers (12) achieved perfect scores on the GFMT, with another 13 super recognisers making only one mistake (78% of the sample in total). On the CFMT+, the equivalent figures are 28% making no errors and a further 6% making only 1 error. In contrast, none of the super recognisers scored in this range (max score = 88%) on the OFMT. The OFMT may therefore be more useful than the GMFT or CFMT when attempting to differentiate performance at the highest levels of ability.

To address the perceptual and memory hypotheses, hierarchical multiple regression was used whereby OFMT scores were entered into step 1 of a model predicting CFMT scores, and then group (SR vs control) entered into step 2. The change in *R*^2^ from step 1 to step 2 (.09) was significant (*F*(1,61) = 10.59, *p* < .005), meaning that variance in CFMT scores could be accounted for by group over and above that explained by OFMT scores.

Relationships between all measures included in Study 5 are shown in Table 3. Note that the correlations between the PI-20 and the face processing tasks increase in both Studies 4 and 5 with inclusion of atypical samples (the DP group in Study 4 and the SR group in Study 5), indicating that atypical groups may have an improved insight into their face processing ability. Such a conclusion is consistent with previous work indicating that the majority of the neurotypical population have little insight into their face perception ability, relative to atypical groups (Bobak et al., 2019).

**Table 3:** Relationship between all face measures included in Study 5 across all participants. Two asterisks (**) denote a relationship significant at *p* < .01 level

	*n*	*M*	*SD*	Cambridge Face Memory Test (CFMT)	20-Item Prosopagnosia Index (PI-20)	Glasgow Face Matching Task (GFMT)	Oxford Face Matching Test (OFMT)
CFMT	64	60.9	10.8	-			
PI-20	64	36.11	11.4	−.55^**^	-		
GFMT	64	35.7	5.3	.58^**^	−.58^**^	-	
OFMT	64	159.4	12.2	.65^**^	−.60^**^	.65^**^	-

Results from Study 5 supported the validity of the OFMT. A group of individuals independently confirmed as SRs performed significantly better on the test than a matched neurotypical control group. Furthermore, OFMT scores correlated moderately well with CFMT+ scores used, in part, to ‘diagnose’ individuals as SRs. With respect to the question of whether SRs have better face perception or face memory, results were consistent with both improved face perception and improved face memory in SRs. The SR group were significantly better than the control group on the OFMT, a test with perceptual but not memory demands. The SR group were also significantly better than the control group on the CFMT, a task with perceptual and memory demands, and this improvement in performance could not be fully accounted for by the superior perceptual ability identified with the OFMT (as demonstrated using the hierarchical multiple regression analysis). Results therefore suggest that, as a group, individuals with SR have superior abilities both to form perceptual representations of faces and to remember facial identities. It is worth noting that these results do not suggest the existence of two subgroups within this population, with only three of the SRs scoring below the neurotypical mean on the perceptual task, while the rest show superior performance on both the perceptual task (OFMT) and the memory task (CFMT+).

## General discussion

Tests of facial identity recognition are difficult to calibrate for the entire range of performance (from individuals with DP, through the normal range of performance, to individuals who are classified as SRs). In order to be sensitive to individual differences across a wide range of performance, test items need to be of varying difficulty—with easier items allowing sensitivity to individual differences within the DP and lower end of typical performance range, and more challenging items allowing sensitivity within the SR and higher end of typical performance range. A potential problem arises, however, when assessing difficulty. Given that face processing is a task that may be carried out using a number of different strategies—and the propensity to use different strategies may vary across groups of interest such as DPs, SRs, or individuals with Autism Spectrum Disorder—item difficulty should be established in a way that is not biased towards one particular population. The solution adopted here was to use algorithmic judgements of similarities of face pairs to construct items of a range of difficulties. These stimuli were used to develop the OFMT, a test of face perception.

The reliability and validity of the OFMT was assessed across five studies. The range of performance observed in neurotypical participants in Study 1 suggested that the OFMT is sensitive to individual differences within this population, with no participant performing at chance levels, or scoring 100% accuracy. The OFMT demonstrated a small-to-moderate correlation with the current gold-standard test of face recognition, the CFMT. This degree of correlation is encouraging, as the CFMT is thought to measure both face perception and memory, whereas the OFMT is thought to measure face perception only. One would therefore expect a modest degree of correlation between performance on the two measures, but not very large correlations. A correlation of similar size was observed between the long and short forms of the OFMT, suggesting that the short form currently provides only a very noisy indicator of long-form performance. The degree of correlation suggests further work is required to make the short form a valid proxy for long-form performance. Until the short form of the OFMT is revised, the short form of the GFMT may be preferable for use when testing time is restricted. Finally, Study 1 established that algorithmic similarity judgements were in accordance with judgements provided by neurotypical participants, and that correlations were of greater magnitude for face images taken from the same person compared to face images of different individuals, replicating previous findings (Hancock et al., 2020). The size of these correlations was moderate, supporting the contention that the algorithms are not simply mimicking the strategy used by neurotypical participants when judging similarity. This latter result supports the basic premise behind using algorithmic ratings rather than ratings provided by a large group of neurotypical raters. The findings of Study 2 replicated those of Study 1, despite Study 2 being administered online rather than in the laboratory like Study 1. This replication suggests that the OFMT is suitable for remote administration and scalable to large numbers of participants. In addition, Study 2 supported the hypothesised relationship between the similarity of face images and difficulty of items.

Further validation of the OFMT was achieved by Studies 4 and 5, which demonstrated that a group of DPs performed worse, and a group of SRs performed better, than matched neurotypical control groups. The substantial correlations between OFMT and CFMT within the DPs and SRs also suggest that the OFMT is a sensitive measure of individual differences in face perception across the entire range of performance. In addition to validating the OFMT, findings contribute to the debate concerning the nature of atypical face recognition in DPs and SRs, particularly whether it is face perception or face memory that is atypical. The conclusion from both studies was that, at the group level, both DPs and SRs have atypical face perception and face memory. Such a conclusion is in accordance with recent studies addressing this issue in DP (see Biotti et al., 2019). A wide range of performance was observed in both DPs and SRs, with some, although few, participants scoring in the typical range.

It should be noted, however, that it is an oversimplification to describe the OFMT as a pure measure of face perception and the CFMT as a measure of face perception plus face memory. Both tests also require the ability to match two instances of an individual’s face, or to recognise the mismatch between faces of different individuals. This face matching stage must occur following the construction of a perceptual representation of the face. In practice, face matching is required by both tests, but the face matching requirements of the OFMT are greater than those of the CFMT as the potential differences between images of the same individual’s face, and the similarity of different individuals’ faces, are likely greater in the OFMT than CFMT. While the greater matching requirement of the OFMT does not invalidate the conclusions of Studies 4 and 5, this comparison of tasks does highlight that both face perception and face matching ability are required for successful performance on the OFMT. Here, face matching ability refers to the accuracy of representation of (1) the degree of difference, and (2) the kinds of difference permissible, between two facial images before they must be judged as faces of different individuals.

Finally, Study 3 assessed the reliability of the OFMT and compared it to two other standard tests of face processing, the CFMT and GFMT. Reliability data on these tests is surprisingly scarce, given the age of the tests and their extensive use, but data from Study 3 indicated that the reliability of the OFMT is good, and statistically indistinguishable from the CFMT and GFMT. Thus, Studies 1–5 suggest the OFMT is a valid and reliable measure of face perception.

The key distinction between the OFMT and existing tests of face processing is the use of facial recognition algorithms to obtain an objective measure of face similarity. This has a number of advantages, most notably that similarity values are not biased against particular sub-populations, whose strategies used for recognising faces may differ from those of the neurotypical majority. A further advantage is practical: the use of algorithmic similarity judgements means that, provided adequate stimulus material exists, multiple parallel forms of the OFMT can be generated to allow repeated testing with independent stimuli. With appropriate selection of stimuli for algorithmic similarity rating, these parallel forms could be specific to processing of faces of certain ethnicities, ages, or genders. At least two potential disadvantages exist, however. The first is that any particular algorithm may use a unique strategy to assess similarity, meaning that algorithmic similarity ratings do not necessarily match those produced by any group of human raters. Data reported here show a moderate correlation between algorithmic ratings and human raters, potentially because three different algorithms were used to obtain algorithmic similarity ratings, which were then averaged, and stimulus pairs which produced discrepant ratings among the algorithms were not included. Therefore, while the algorithmic values used in the OFMT appear to have provided a valid, as well as objective, measure of similarity, separate processes are likely to contribute to these values across different algorithms and human perceivers. The second potential disadvantage is that algorithms may be systematically worse at judging the similarities of particular groups of faces (e.g., faces of particular gender, ethnicity, age, or their interaction). Despite numerous press assertions that this may be so, official testing by the US National Institute of Standards and Technology suggests that the 17 most accurate algorithms had similar levels of accuracy across demographic groups (McLaughlin & Castro, 2020). However, the assumption of non-biased performance by stimulus class is one that should be continuously scrutinised as facial recognition algorithms develop.

Another feature of the OFMT that distinguishes it from most other tests of face processing is that relatively naturalistic face images are used. While static, faces in the OFMT are shown with hair and without cropping any information about the face shape. Difficulty on more challenging trials is not introduced through introduction of artificial Gaussian noise or partial occlusion of facial information. Rather, difficulty is increased in ways in which it occurs in life—matching facial images of the same person when their appearance has changed substantially, as well as distinguishing similar-looking people from one another. The OFMT is therefore arguably more ecologically valid than existing tasks of face recognition, with clear applications to real-life situations such as identity verification and forensic contexts.

In summary, this paper describes the development and validation of the OFMT, a non-biased test of face perception, which is suitable for use across the full range of face perception ability. Using this task, along with the CFMT, which assesses both face perception and face memory, results indicate that the atypical face recognition in DPs and SRs is a product of both atypical face perception and face memory. The OFMT is freely available to researchers on the Gorilla Open Materials repository (https://gorilla.sc/openmaterials) for non-commercial use.

## Context

The OFMT was designed to provide a measure of face perception which is not biased towards any particular group. We wanted to avoid designing a test in which atypical groups (for example individuals with autism) might do poorly simply because the difficulty of the test was better calibrated for typical individuals. We therefore used facial recognition algorithms to select stimuli of varying difficulty—providing a test of face perception that is not biased towards any one group and is sensitive to individual differences across the full range of human performance. The test can be administered online, and results suggest it is reliable and valid. We used this test to show that individuals who have very poor face recognition ability tend to be bad at both perceiving faces and remembering faces, while those who have very good face recognition are good at both perceiving faces and remembering faces. This work continues our laboratory’s exploration of individual differences in identity and emotion perception in typical and atypical groups.

## Supplementary Information

ESM 1 (DOCX 16 kb)

## Footnotes

1 Here ‘objective*’* is used as defined in the Oxford English Dictionary, “not influenced by personal feelings or opinions in considering and representing facts; impartial”, without implying accuracy or status as ‘ground truth’.
