# Supplementary material for: The Oxford Face Matching Test: A non-biased test of the full range of individual differences in face perception
Source: Behav Res Methods. 2021 Jun 15;54(1):158–73. doi: 10.3758/s13428-021-01609-2 (PMC8863687; doi:10.3758/s13428-021-01609-2)
Supplement: Supplementary file 1 — (DOCX 16 kb) [file 13428_2021_1609_MOESM1_ESM.docx]

**Supplemental Files**

For each neurotypical participant in Studies 1–5 (Studies 1–3: all participants, Studies 4 and 5: neurotypical controls), accuracy on match (‘same’) and mismatch (‘different’) trials were separately calculated. Data are reported here across all experiments. Correlations between accuracy on match and mismatch trials, and between those two measures and other tests of face memory and perception, are also included. Note that the GFMT was not included in Studies 1 and 2, so the correlation between performance on the GFMT and other measures includes fewer participants (see Ns in Table 1S).

For both types of trials, performance varied greatly (match: 31–98%; mismatch: 23–97%), though the performance on mismatch trials (*M* = 75.26, *SD* = 12.05) was significantly better than performance on match trials (*M* = 72.34, *SD* = 12.36), *t*(218) = 92.42, *p* < .001.

Relationships across all measures are reported in Table 1S.

Table 1S. Relationships between all face processing measures included across studies, including separate accuracy metrics for match (‘same’) and mismatch (‘different’) trials. Correlations significant at the 0.01 level are denoted with two asterisks (**).

|  | *n* | Match (‘same’) trials | Mismatch (‘different’) trials | Oxford Face Matching Test (OFMT) | Cambridge Face Memory Test (CFMT) | Glasgow Face Matching Task (GFMT) |
| --- | --- | --- | --- | --- | --- | --- |
| *Match* | 286 | - |  |  |  |  |
| *Mismatch* | 286 | -.55** | - |  |  |  |
| OFMT | 286 | .46** | .41** | - |  |  |
| CFMT | 286 | .20** | .19** | .43** | - |  |
| GFMT | 286 | .12 | .36** | .52** | .52** | - |
